# Supplementary material for: Diatom-Inspired Structural Adaptation According to Mode Shapes: A Study on 3D Structures and Software Tools
Source: Biomimetics (Basel). 2024 Apr 18;9(4):241. doi: 10.3390/biomimetics9040241 (PMC11047921; doi:10.3390/biomimetics9040241)
Supplement: Supplementary file 1 [file biomimetics-09-00241-s001.zip › biomimetics-2898203-supplementary.pdf]

Supplementary Material

S1: Mesh convergence study results for the investigated solid structures

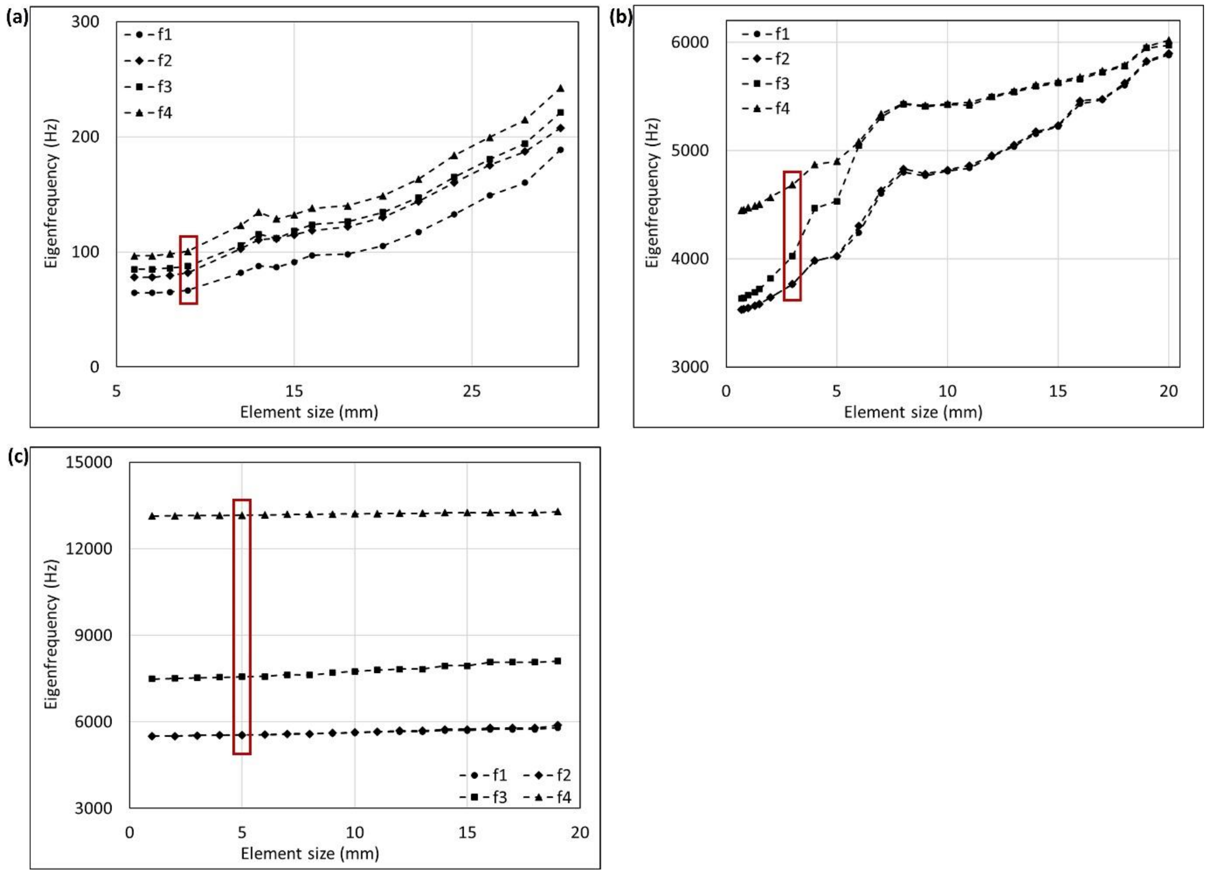

Figure S1: Volume mesh convergence study results for (a) the hollow cuboid, (b) the thick hexagonal prism, and (c) the cube. The chosen element size is framed in red.

S2: Mesh convergence study results for the investigated shell structures

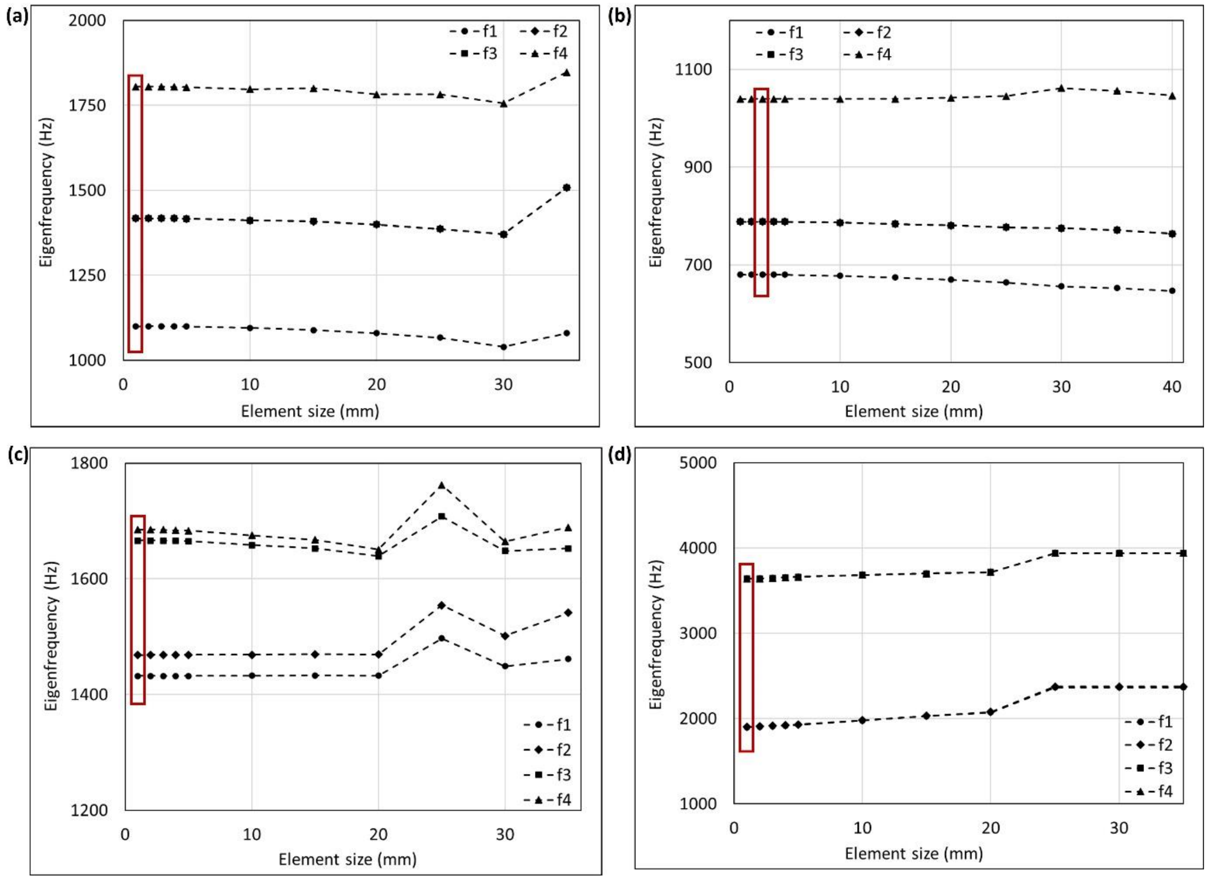

Figure S2: Shell mesh convergence study results for (a) the truncated pyramid, (b) the thin hexagonal prism, (c) the curved rectangular duct, and the connector square. The chosen element size is framed in red.

S3: Mesh convergence study results for the investigated beam

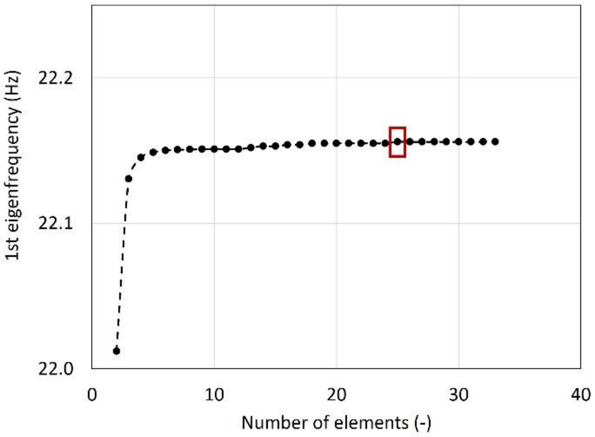

Figure S3: Mesh convergence study of the beam

S4: Results of the mode shape adaptation according to mode 2 applied to the studied 3D structures

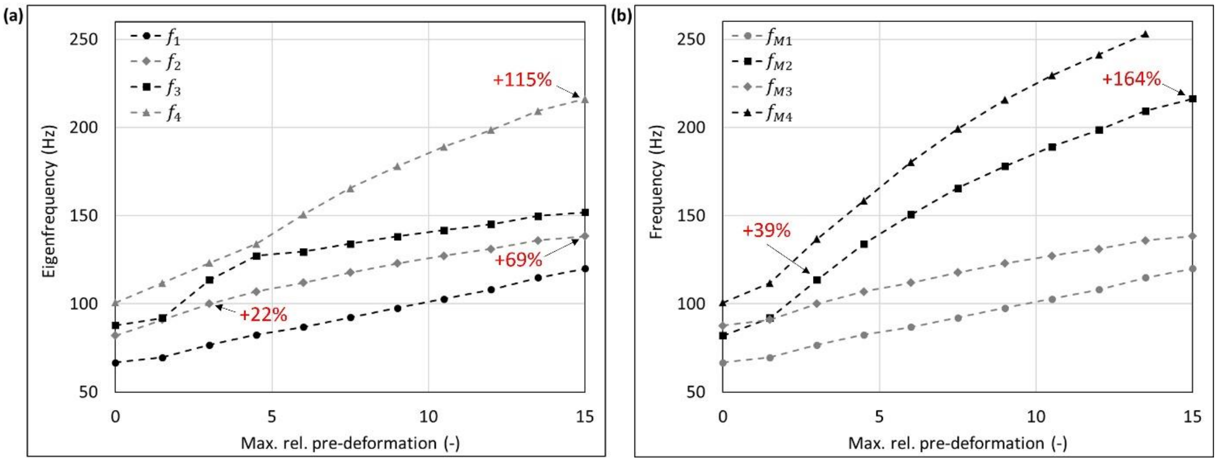

Figure S4.1: Eigenfrequencies f_1 to f_4 (a) and mode shape frequencies f_{M1} to f_{M4} (b) of the hollow cuboid pre-deformed according to mode 2, considering different maximum relative pre-deformations. For two pre-deformations, the frequency deviation compared to the undeformed structure of the mode shape adapted to the structure is given in red. In addition, the maximum obtained frequency increase is also noted.

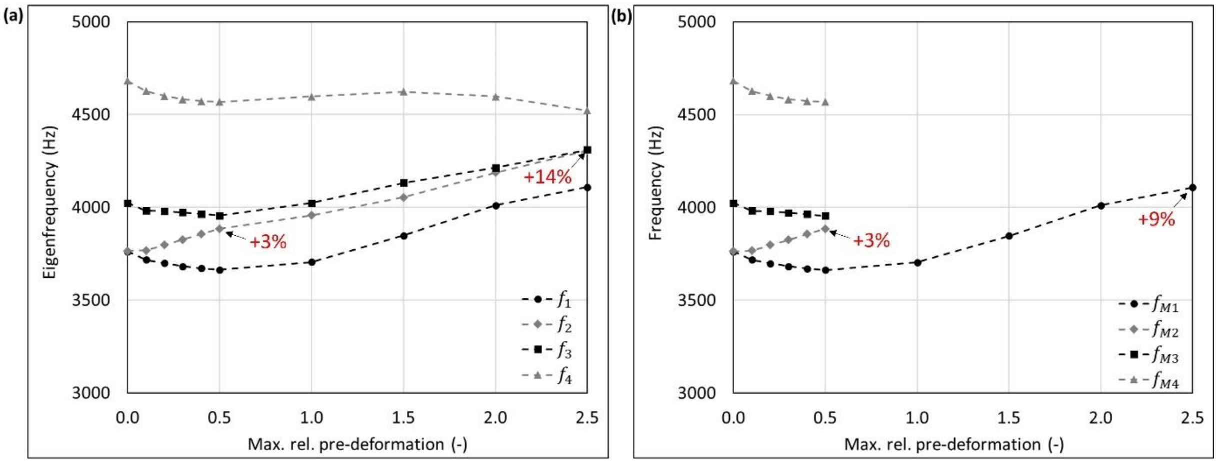

Figure S4.2: Eigenfrequencies f_1 to f_4 (a) and mode shape frequencies f_{M1} to f_{M4} (b) of the thick hexagonal prism pre-deformed according to mode 2, considering different maximum relative pre-deformations. For two pre-deformations, the frequency deviation compared to the undeformed structure of the mode shape adapted to the structure is given in red. In addition, the maximum obtained frequency increase is also noted.

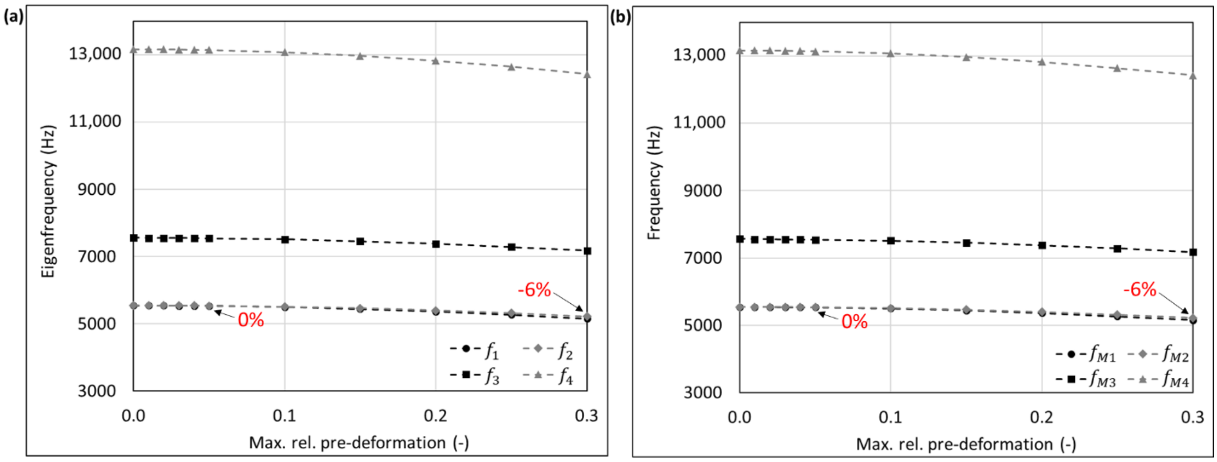

Figure S4.3: Eigenfrequencies f_1 to f_4 (a) and mode shape frequencies f_{M1} to f_{M4} (b) of the cube pre-deformed according to mode 2, considering different maximum relative pre-deformations. For two pre-deformations, the frequency deviation compared to the undeformed structure of the mode shape adapted to the structure is given in red. In addition, the maximum obtained frequency increase is also noted.

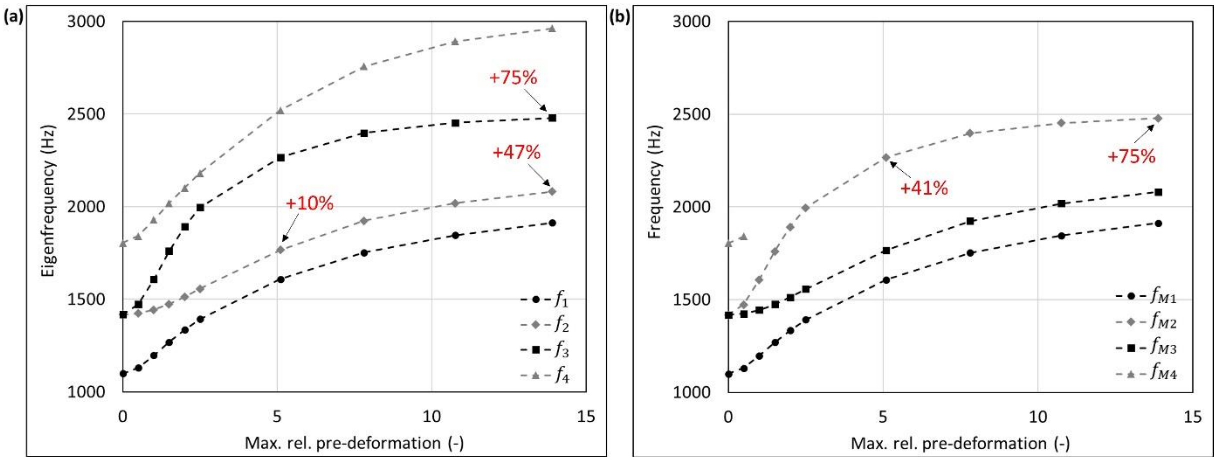

Figure S4.4: Eigenfrequencies f_1 to f_4 (a) and mode shape frequencies f_{M1} to f_{M4} (b) of the truncated pyramid pre-deformed according to mode 2, considering different maximum relative pre-deformations. For two pre-deformations, the frequency deviation compared to the undeformed structure of the mode shape adapted to the structure is given in red. In addition, the maximum obtained frequency increase is also noted.

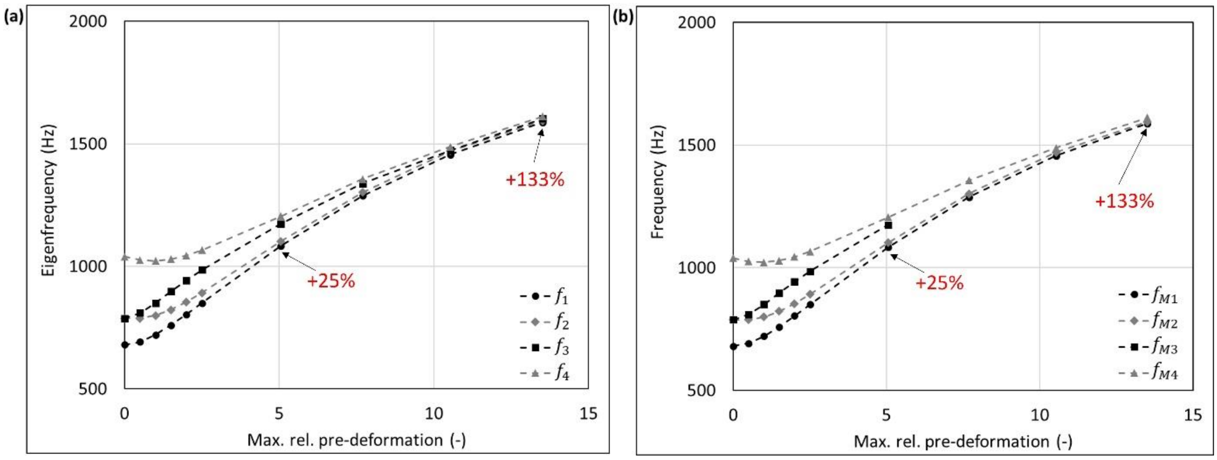

Figure S4.5: Eigenfrequencies f_1 to f_4 (a) and mode shape frequencies f_{M1} to f_{M4} (b) of the thin hexagonal prism pre-deformed according to mode 2, considering different maximum relative pre-

deformations. For two pre-deformations, the frequency deviation compared to the undeformed structure of the mode shape adapted to the structure is given in red. In addition, the maximum obtained frequency increase is also noted.

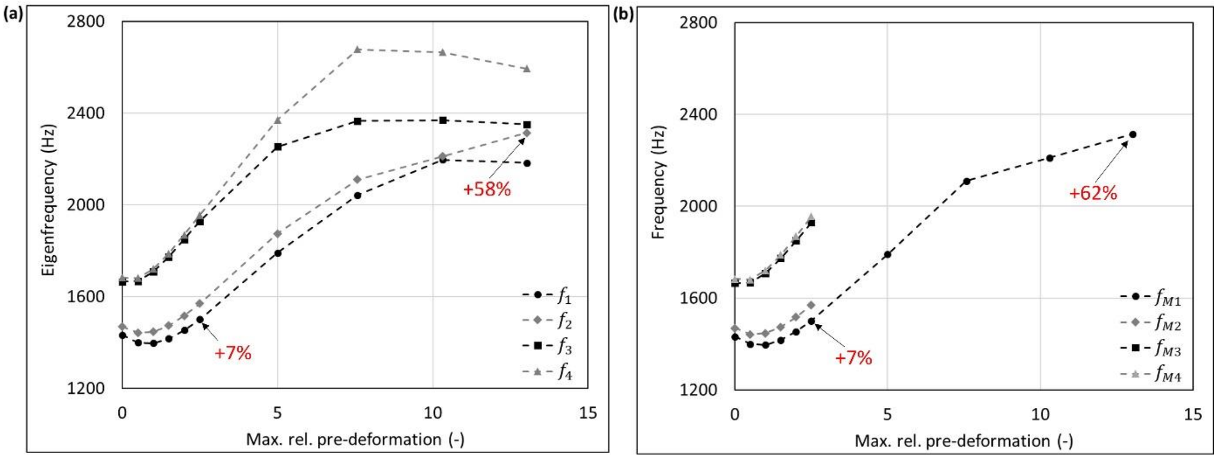

Figure S4.6: Eigenfrequencies f_1 to f_4 (a) and mode shape frequencies f_{M1} to f_{M4} (b) of the curved rectangular duct pre-deformed according to mode 2, considering different maximum relative pre-deformations. For two pre-deformations, the frequency deviation compared to the undeformed structure of the mode shape adapted to the structure is given in red. In addition, the maximum obtained frequency increase is also noted.

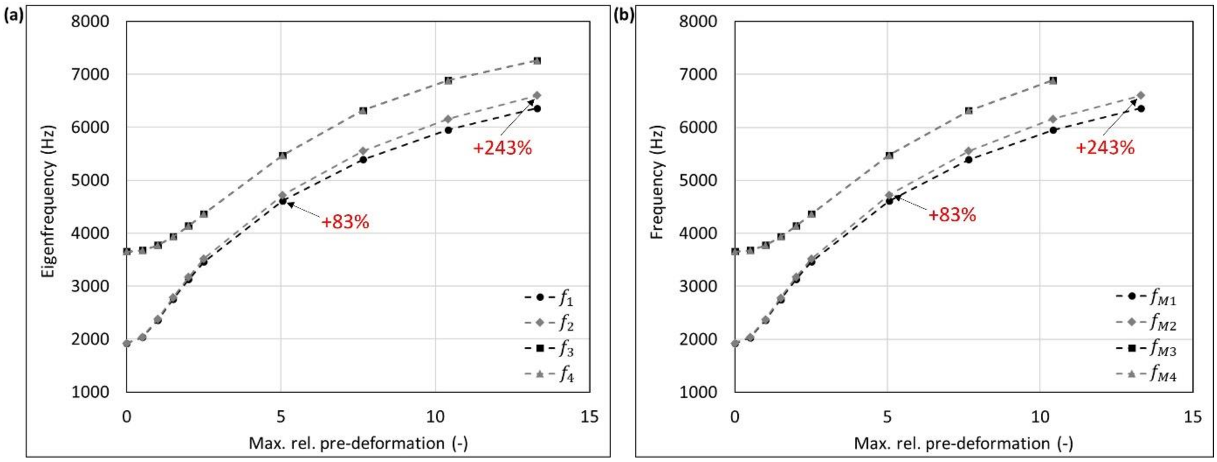

Figure S4.7: Eigenfrequencies f_1 to f_4 (a) and mode shape frequencies f_{M1} to f_{M4} (b) of the connector square pre-deformed according to mode 2, considering different maximum relative pre-deformations. For two pre-deformations, the frequency deviation compared to the undeformed structure of the mode shape adapted to the structure is given in red. In addition, the maximum obtained frequency increase is also noted.
